# Division of Labor Regulates Precision Rescue Behavior in Sand-Dwelling *Cataglyphis cursor* Ants: To Give Is to Receive

**DOI:** 10.1371/journal.pone.0048516

**Published:** 2012-11-07

**Authors:** Elise Nowbahari, Karen L. Hollis, Jean-Luc Durand

**Affiliations:** 1 Laboratoire d'Éthologie Expérimentale et Comparée (EA 4443), Université Paris 13, Sorbonne Paris Cité, Villetaneuse, France; 2 Interdisciplinary Program in Neuroscience and Behavior, Mount Holyoke College, South Hadley, Massachusetts, United States of America; Université Pierre et Marie Curie, France

## Abstract

Division of labor, an adaptation in which individuals specialize in performing tasks necessary to the colony, such as nest defense and foraging, is believed key to eusocial insects' remarkable ecological success. Here we report, for the first time, a completely novel specialization in a eusocial insect, namely the ability of *Cataglyphis cursor* ants to rescue a trapped nestmate using precisely targeted behavior. Labeled “precision rescue”, this behavior involves the ability of rescuers not only to detect what, exactly, holds the victim in place, but also to direct specific actions to this obstacle. Individual ants, sampled from each of *C. cursor's* three castes, namely foragers, nurses and inactives, were experimentally ensnared (the “victim”) and exposed to a caste-specific group of potential “rescuers.” The data reveal that foragers were able to administer, and obtain, the most help while members of the youngest, inactive caste not only failed to respond to victims, but also received virtually no help from potential rescuers, regardless of caste. Nurses performed intermediate levels of aid, mirroring their intermediate caste status. Our results demonstrate that division of labor, which controls foraging, defense and brood care in *C. cursor*, also regulates a newly discovered behavior in this species, namely a sophisticated form of rescue, a highly adaptive specialization that is finely tuned to a caste member's probability of becoming, or encountering, a victim in need of rescue.

## Introduction

Division of labor, one of the most prominent and widely studied features of colony behavior in social insects [1]–[6], takes one of two general forms: *morphological polyethism*, in which workers' size and/or shape determines what tasks they will perform; and, *temporal polyethism*, in which individuals perform different tasks as they mature [1]–[7]. Temporal polyethism is widespread in social insects and typically follows the pattern of younger workers performing tasks within the nest and older workers performing tasks outside, such as foraging and defense [1]–[7]. Presumably, this behavioral specialization, which is thought responsible for social insects' enormous ecological success, increases the overall efficiency of the colony because workers that focus on and repeat a particular task will perform it more reliably [2], [6]. *Cataglyphis cursor*, a sand-dwelling Mediterranean ant, exhibits temporal polyethism in which foragers, typically the oldest members of the colony, are responsible for securing food, nurses specialize in brood care, and inactives, the youngest workers, remain near the brood but almost never tend them [8], [9].

**Figure 1:**
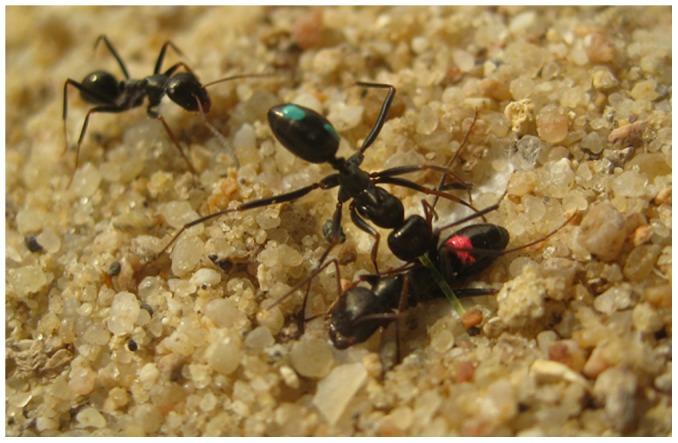
Precision rescue behavior. In addition to digging near an entrapped nestmate and pulling on its limbs, a very simple form of rescue behavior observed in several ant species, *C. cursor* ants somehow are able to identify exactly what obstacle holds the victim in place, to transport sand away from that obstacle, and then to target their bites precisely to it alone, excavating sand as necessary to expose the obstacle further. We have labeled this behavior “precision rescue.” Here, a *C. cursor* rescuer already has transported sufficient sand away from the victim, exposing the nylon thread snare holding its nestmate in place (part of the white filter paper has been exposed as well), and is pictured biting the snare that holds the victim to the paper. Carefully aimed, snare biting never is misplaced, even though the snare has been tied snugly around the pedicel (waist) of the victim and is in direct contact with the victim's body. Photograph by Paul Devienne.

Nowbahari et al. [10] have shown that *C. cursor* ants also are capable of highly sophisticated rescue behavior. That is, when an individual becomes entrapped, as often happens in nature when it is caught under collapsing sand or debris, or falls into a predatory antlion larvae pit [11], [12], nearby nestmates begin by digging near the victim and pulling on its limbs, a very simple form of rescue behavior observed in several ant species [13]–[15]. In addition, however, *C. cursor* rescuers somehow are able to identify exactly what holds the victim in place, to transport sand away from that obstacle, and then, as illustrated in Figure 1, to target their bites precisely to it alone, excavating sand as necessary to expose the obstacle further [10]. Carefully aimed, biting at the obstacle never is misplaced, even though it may be in direct contact with the victim's body.

Subsequent observations of *C. cursor* revealed, however, that not all adults administered help and not all victims were able to elicit help – differences that might reflect other aspects of individuals' division of labor, their physiological maturation or both. That is, because division of labor in *C. cursor* follows an age polyethism pattern [16], *C. cursor* foragers, as in all *Cataglyphis* ant species, are among the older colony members. Foragers, which are capable of high individual nestmate discrimination abilities, are physiologically more mature and, thus, for these reasons we predicted that they would be more likely both to give and to receive aid. Nest-bound inactives, on the other hand, younger individuals that are less physiologically mature, might be less able not only to call for help, but also to provide aid. Finally, nurses, specialized for brood care, might require some of the same behavioral patterns needed by efficient rescuers. Consequently, in the present study, we examined the role of polyethism in the rescue behavior of *C. cursor* ants by conducting tests of rescue behavior in which we systematically varied the caste of both victim and rescuers. Based on their physiological maturation and their other specializations, we predicted that foragers and inactives would differ substantially in their ability both to give and receive aid, with nurses possibly intermediate between these two castes.

As predicted, our results show that temporal polyethism, which controls foraging, defense and brood care in *C. cursor*, also regulates the capacity of these ants to deliver precision rescue behavior. We suggest that this highly adaptive specialization has been finely tuned through evolution to match a caste member's probability of becoming, or encountering, a victim in need of rescue.

## Materials and Methods

### Ethics Statement

Five colonies of *C. cursor*, each with a queen and brood, were collected in Saint-Cyprien and Argelès-sur-Mer, France. These locations were not privately-owned or protected so no specific permission was required. We confirm that the field studies did not involve endangered or protected species.

### Ants and Rearing Conditions

In the laboratory, each colony was housed and maintained separately: A cylindrical closed nest box (15 cm diameter) was connected via a 20-cm plastic tube to an open foraging area, namely a plastic tray (28 cm×27.5 cm×8.5 cm high) covered with a thin layer of sand. Ants were fed mealworm larvae and an apple-honey mixture twice per week. The colony room was maintained at 28±2°C, 20 to 40% humidity, with a 12∶12 light∶dark cycle. In each of the 5 colonies, 135 ants were identified as foragers, nurses, or inactives (45 individuals per caste) for a total of 675 individuals; ants were marked on the thorax with a distinct spot of indelible paint (Uni Paint Marker PX 20, Mitsubishi Pencil Co., LTD). Experiments were conducted during ants' active period, between 09:00h and 14:00h, from February to May, when broods were present, which results in marked polyethism [8], [9].

### Procedure

We conducted tests of rescue behavior in which we systematically varied the caste of both victim and rescuers in a 3×3 factorial design. That is, each test consisted of a group of 5 potential rescuers of the same caste obtained from the same colony – either 5 foragers, 5 nurses, or 5 inactives – paired with a single experimentally ensnared victim, either forager, nurse, or inactive, for a total of 9 different rescuer-victim combinations. To insure reliability, we recorded 15 independent observations for each rescuer-victim combination, namely 3 samples from each of 5 different *C. cursor* colonies, resulting in 135 separate tests. In addition, for each of the 9 rescuer-victim combinations, 15 control tests (i.e., 3 per colony) were conducted with the same rescuer-victim combination, but in which the victim was anesthetized by chilling, a control that, in previous work [10], did not elicit any rescue behavior whatsoever.

To conduct each test, we followed the testing procedures described in Nowbahari et al. [10]. Briefly, a plastic ring, which was used to confine rescuers for testing, was placed close to the nest entrance and the ant victim was prepared by tying it to a small piece of filter paper. Following preparation of the victim, 5 marked subjects were chosen at random from a single caste within a colony (i.e., foragers, nurses, or inactives) and placed inside the ring for 2 min, allowing them to habituate to having been moved, and to the ring itself. We used the group of 5 nestmates per trial because previous work showed this procedure resulted in reliable rescue behavior [10]. Next, the filter paper containing the victim was inserted in the center of the ring and covered with a thin layer of sand, such that the victim's head and thorax, but not the filter paper, was visible. Following the 4-min test, the victim was removed and the ring was lifted, freeing ants to return to the nest or to remain in the foraging area. Each group of 5 rescuers was tested with an active victim of a particular caste, as well as with another, different victim of the same caste that had been anesthetized by chilling (2 min at −4C°), rendering it motionless. The order of these two tests, namely with an active or anesthetized victim, was counterbalanced within each colony, as well as within each victim-rescuer combination. Marking insured that no victim was tested twice. A new snare and filter paper were used for each test.

### Statistical Analysis

For each test of rescue behavior, the group of 5 potential rescuers constituted the statistical unit of analyses. That is, although marking enabled us to record each ant's behavior separately, the dependent variable was the duration of rescue behavior summed across each of the 5 ants during 4 minutes of observation, or, in the case of latency data, the latency of the first act of rescue by any one of the 5 rescuers. Nonparametric statistical tests were used to analyze the data (StatXact 8, Cytel 2007). To compare the 3 castes, permutation tests for K independent samples were used. Whenever the overall comparison of the three castes was significant, we analyzed the three paired comparisons with permutation tests for two independent samples, using the Bonferroni-Holm correction (noted below as P' for each adjusted P-value). Permutation tests also were used to examine the correlation between latency and duration measures. Because we did not find any statistical differences in rescue behavior between the 5 different ant colonies (Permutation test for K independent samples, all *P*>0.55), we combined the results across the 5 colonies. Finally, although we report the results of permutation tests for all analyses, we obtained exactly the same pattern of significant and non-significant results using Kruskal-Wallis and Mann-Whitney *U* tests.

## Results

### Duration of Rescue Behavior

The duration of rescue behavior differed significantly between the three castes of rescuers (P<0.0001). Overall, foragers, whose mean duration of rescue was 180.4±25.9 s, helped significantly longer than nurses (81.3±15.3 s) (P' = 0.001), and both foragers and nurses helped significantly longer than inactives (2.6±1.1 s) (both P's<0.0001). Indeed, when the group of five rescuers was composed of inactives, rescue behavior was rare.

The duration of rescue behavior also differed significantly between the three castes of victims (P<0.0001). Overall, forager and nurse victims were helped significantly longer than inactives (P'<0.0001 and P' = 0.0002). The mean duration of rescue received by foragers and nurses was 140.7±24.9 s and 102.1±20.2 s respectively, whereas inactives received help for only 21.4±8.2 s. We found no significant difference between forager and nurse victims (P' = 0.24).

**Figure 2:**
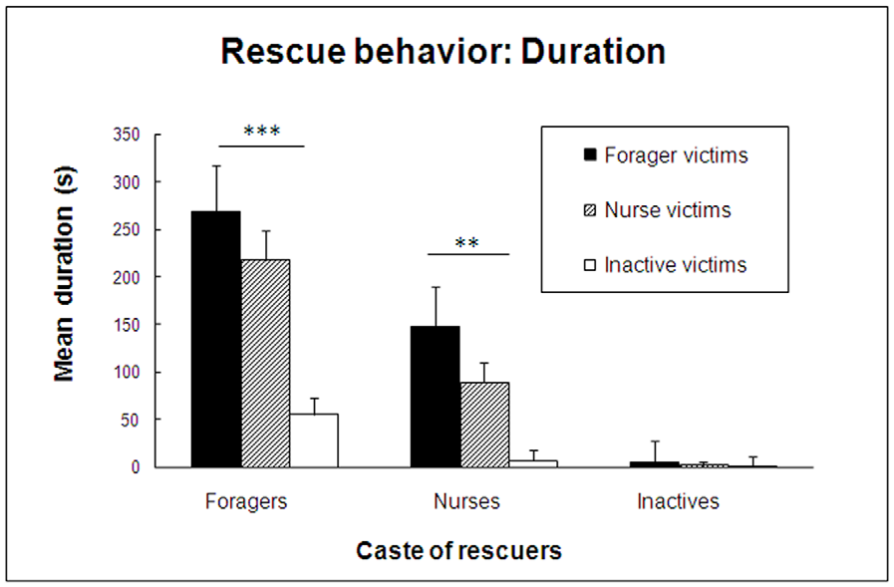
Duration of rescue behavior. Mean duration (± SE) of rescue behavior performed by a group of five *C. cursor* rescuer, all foragers, all nurses or all inactives, in the presence of a single experimentally ensnared victim, either a forager, a nurse or an inactive.

As Figure 2 shows, the patterns of rescue behavior delivered and received by the three castes also differed substantially. Forager rescuers did not dispense aid identically to the three castes of victims (P' = 0 0009), delivering significantly more help to forager and nurse victims than to inactives (P' = 0.0004 and P' = 0.002, respectively). However, foragers helped forager and nurse victims equally (P = 0.43). Nurse rescuers displayed an identical pattern of preferential treatment (P = 0.0002), delivering significantly more help to forager and nurse victims than to inactives (P'<0.0001 and P' = 0.0003, respectively), but treating forager and nurse victims similarly (P' = 0.13). When, however, the rescuer group was composed of members of the inactive caste, victims were mostly ignored, with no significant difference between the three castes of victims (P = 0.13).

Finally, analysis of the duration of snare biting, the precision rescue behavior previously reported by Nowbahari et al. [10], revealed a significant difference between groups (P'<0.0001). Foragers engaged in snare biting for a significantly longer duration than did nurses (P' = 0.01) and inactives (P'<0.0001), and nurses spent more time snare biting than did inactives (P' = <0.0001). This particular behavior, which might reasonably be argued to represent a measure of workers' efficiency as it is directed at the object actually holding the victim in place, lasted for 14.8±3.8 s in foragers, but for only 4.4±1.0 s in nurses; inactives displayed no snare biting whatsoever.

### Latency of Rescue Behavior

**Figure 3:**
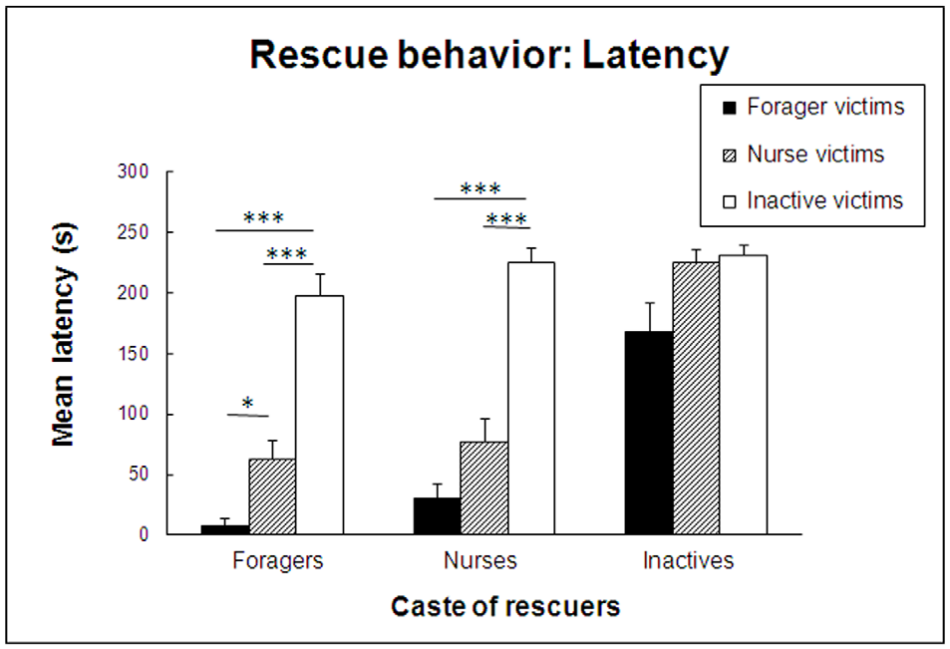
Latency of rescue behavior. Mean latency (± SE) of the first act of rescue behavior performed by any member of the group of five *C. cursor* rescuers, all foragers, all nurses or all inactives, in the presence of a single experimentally ensnared victim, either a forager, a nurse or an inactive.

The latency to rescue victims revealed a virtually identical pattern of results. Overall, latency to rescue differed significantly across the three castes of rescuers (P<0.0001), with foragers engaging in rescue behavior significantly sooner than nurses (P' = 0.01), and both foragers and nurses responding significantly sooner than inactives (both P's<0.0001). Likewise, as Figure 3 shows, the victim's caste had a large effect on the latency of rescue, differing significantly across the three castes of victims (P<0.0001). Specifically, both foragers and nurses were helped significantly faster than inactives (both P's<0.0001); however, forager and nurse victims received help equally quickly (P' = 0.28). As expected, control tests with anesthetized victims elicited no response whatsoever in 133 of 135 tests. In two tests of the forager-forager combination, rescue occurred for a few seconds, followed by complete abandonment of the victim.

### Correlation between Duration and Latency of Rescue Behavior

Finally, statistical analysis revealed a significant negative correlation between the latency of the first act of rescue behavior and the total duration of rescue behavior. In particular, rescue behavior occurred more quickly, and persisted longer, when the rescuers were either foragers or nurses and the victim was either a forager or a nurse (r = −0.50, P = 0.0002).

## Discussion

Our data reveal a novel behavioral specialization in a eusocial insect, a specialization never before reported in the literature, either in ants or in any other eusocial insect. In support of our hypothesis, the expression of specialized rescue behavior in *C. cursor* is characterized by a form of temporal polyethism in which, as workers mature and assume the duties of nurses and foragers, they are more likely to respond quickly to a nestmate in distress and to persist in that behavior for a longer time. Furthermore, our results suggest that caste membership determines not only the ability to provide aid, but also to receive it. That is, foragers were able both to administer, and to obtain, the most help; inactives were incapable of responding to victims, as well as incapable of eliciting help from potential rescuers, regardless of caste; and, nurses generally performed intermediate levels of aid, mirroring their intermediate caste status.

According to Retana and Cerdá [8], division of labor in *C. cursor* adult workers is based on a well-defined temporal polyethism in which young workers are initially inactive, and then, as they mature, perform various nest-related tasks, and finally leave the nest to become foragers. This same pattern is exhibited by several other *Cataglyphis* species, including *Cataglyphis bicolor* [17], and *Cataglyphis niger* [18]. The differences we observed in *C. cursor* ants' ability to rescue nestmates map easily on this pattern of temporal polyethism, a pattern that places some castes of workers at greater risk of entrapment and, thus, in greater need of the capacity to give and receive aid.

For example, *C. cursor* foragers, like other *Cataglyphis* ants, do not form ant trails to food, but search individually, relying on their highly-developed orientation abilities [1], [19]–[22]. Under the hot desert conditions experienced by these ants, entrapment easily could be lethal. Because foragers are the sole providers of food, but represent only 14.6% of the workers [8], a trapped forager represents a potentially large cost to the colony. Natural selection can ameliorate this cost, however, through specialized rescue behavior, a mechanism that enables foragers both to call for help, and to respond to the call of another forager.

Like foragers in some ways, nurses also must respond to nestmates, in their case larvae, frequently moving them using nearly the same pulling behavior involved in rescue [1], [8]. Thus, nurses' ability to rescue a trapped nestmate is not surprising. Nonetheless, our results show that they are not as expert in rescue behavior as foragers, a difference that could reflect their slightly less mature development, their inexperience with trapped nestmates, or both. Finally, because inactives, the youngest workers, also never leave the nest but, unlike nurses, have no responsibility for brood care, they have no need of a capacity to rescue nestmates.

This age-dependent division of labor in *C. cursor* almost certainly reflects workers' physiological maturation, including both brain development, as has been demonstrated in another *Cataglyphis* species, *C. albicans* [23], as well as glandular development [3]. For example, in *Myrmica rubra* ant workers, the volume of secretions produced by the Dufour and poison glands, which are used to signal alarm, increases with the age of workers [24]. In a study of *C. cursor* pheromones, we found some evidence that these same two glands are involved in rescue behavior (unpublished data). Thus, because *C. cursor* foragers are the oldest workers, they would be expected to possess more developed glands, which would enable them to emit a more intense alarm signal than less-developed nurses; in turn, nurses would be expected to signal more strongly than even less-developed inactives. Because, in the current study, the ability to receive aid from rescuers – which reflects ants' ability to signal their distress – generally matched their ability to provide aid to victims, our results suggest that both sending and perceiving the distress signal develops concurrently.

In sum, our study shows that precision rescue behavior, a highly developed and complex behavior in which *C. cursor* ants somehow are able to identify exactly what holds an entrapped nestmate in place and then to target their behavior to it alone, is regulated by temporal polyethism, a form of division of labor in which adult workers perform different tasks as they mature. Although several anecdotal reports of rescue behavior exist in the scientific literature [25], the ability to perform specifically targeted rescue behavior – what we call precision rescue – has been studied experimentally in only two species, namely ants [10] and, very recently, in rats [26]. Although researchers have yet to determine why some species possess this capability and others do not, the ability of *C. cursor* to rescue its nestmates appears to have evolved to meet the particular risks it faces in its harsh environments.

## References

[1] Hölldobler B, Wilson EO (1990) The ants. Cambridge, MA: Harvard University Press. 732 p.

[2] Beshers SN, Fewell JH (2001) Models of division of labor in social insects. Ann Rev Entomol 46: 413–440. 11112175 10.1146/annurev.ento.46.1.413

[3] Robinson GE (1992) Regulation of division of labor in insect societies. Ann Rev Entomol 37: 637–665. 1539941 10.1146/annurev.en.37.010192.003225

[4] Gordon DM (1999) Ants at work: How an insect society is organized. New York: Simon and Schuster. 182 p.

[5] Gordon DM (2010) Ant encounters: Interaction networks and colony behavior. Princeton, NJ: Princeton University Press. 184p.

[6] Oster GF, Wilson EO (1978) Caste and ecology in the social insects. Princeton, NJ: Princeton University Press. 372 p. 740003

[7] Robinson GE, Page RE Jr, Huang ZY (1994) Temporal polyethism in social insects is a developmental process. Anim Behav 48: 467–469.

[8] Retana J, Cerdá X (1990) Social organization of *Cataglyphis cursor* ant colonies (Hymenoptera: Formicidae): inter- and intraspecific comparisons. Ethology 84: 105–122.

[9] Retana J, Cerdá X (1991) Behavioural variability and development of *Cataglyphis cursor* ant workers (Hymenoptera, Formicidae). Ethology 89: 275–286.

[10] Nowbahari E, Scohier A, Durand J-L, Hollis KL (2009) Ants, *Cataglyphis cursor*, use precisely directed rescue behavior to free entrapped relatives. PLoS ONE 4 (8) e6573. 19672292 10.1371/journal.pone.0006573 PMC2719796

[11] Czechowski W, Godzińska EJ, Kozłowski MW (2002) Rescue behavior shown by workers of *Formica sanguinea* Latr., *F. fusca* L. and *F. cinerea* Mayr (Hymenoptera: Formicidae) in response to their nestmates caught by an ant lion larva. Ann Zool 52: 423–431.

[12] Hollis KL, Cogswell H, Snyder K, Guillette LM, Nowbahari E (2011) Specialized learning in antlions (Neuroptera: Myrmeleontidae), pit-digging predators, shortens vulnerable larval stage. PLoS ONE 6 (3) e17958. 21479229 10.1371/journal.pone.0017958 PMC3066215

[13] Wilson EO (1958) A chemical releaser of alarm and digging behavior in the ant *Pogonomyrmex badius* (Latreille). Psyche 65: 41–51.

[14] Blum MS, Warter SL (1966) Chemical releasers of social behavior. VII. The isolation of 2-heptanone from *Conomyrma pyramica* (Hymenoptera: Formicidae: Dolichoderinae) and its modus operandi as a releaser of alarm and digging behavior. Ann Entomol Soc Am 59: 774–779.

[15] Spangler HG (1968) Stimuli releasing digging behavior in the western harvester ant (Hymenoptera: Formicidae). J Kansas Entomol Soc 41: 318–323.

[16] Mayade S, Cammaerts M-C, Suzzoni J-P (1993) Home-range marking and territorial marking in *Cataglyphis cursor* (Hymenoptera: Formicidae). Behav Proc 30: 131–142. 10.1016/0376-6357(93)90003-A 24896716

[17] Schmid-Hempel P, Schmid-Hempel R (1984) Life duration and turnover of foragers in the ant *Cataglyphis bicolor* (Hymenoptera, Formicidae). Ins Soc 31: 345–360.

[18] Nowbahari E, Fénéron R, Malherbe M-C (2000) Polymorphism and polyethism in the formicinae ant *Cataglyphis niger* (Hymenoptera). Sociobiology 36: 485–496.

[19] Wehner R, Harkness RD, Schmid-Hempel P (1983) Foraging strategies in individually searching ants, *Cataglyphis bicolor* (Hymenoptera: Formicidae). Akad Wiss Lit Mainz Math Naturwiss Kl. Stuttgart: Verlag. 79 pp.

[20] Wehner R (1987) Spatial organization of foraging behavior in individually searching desert ants, *Cataglyphis* (Sahara Desert) and *Ocymyrmex* (Namib Desert). In: Pasteels JM, Deneubourg JL, editors. From individual to collective behavior in social insects. Basel: Birkhäuser. pp. 15–42.

[21] Lenoir A, Aron S, Cerdá X, Hefetz A (2009) *Cataglyphis* desert ants: a good model for evolutionary biology in Darwin's anniversary year – A review. Isr J Entomol 39: 1–32.

[22] Fourcassie V, Dahbi A, Cerdá X (2000) Orientation and navigation during adult transport between nests in the ant *Cataglyphis iberica* . Naturwissenschaften 87: 355–359. 11013887 10.1007/s001140050739

[23] Seid MA, Wehner R (2009) Delayed axonal pruning in the ant brain: a study of developmental trajectories. Dev Neurobiol 69: 350–364. 19263416 10.1002/dneu.20709

[24] Cammaerts-Tricot M-C (1974) Production and perception of attractive pheromones by differently aged workers of *Myrmica rubra* (Hymenoptera - Formicidae). Insec soc 21: 235–248.

[25] Nowbahari E, Hollis KL (2010) Rescue behavior: Distinguishing between rescue, cooperation, and other forms of altruistic behavior. Communicative and Integrative Biology 3: 77–79. 20585494 10.4161/cib.3.2.10018 PMC2889958

[26] Bartal IB-A, Decety J, Mason P (2011) Empathy and pro-social behavior in rats. Science 334: 1427–1430. 22158823 10.1126/science.1210789 PMC3760221

